# The Immunomodulatory Potential of Wharton's Jelly Mesenchymal Stem/Stromal Cells

**DOI:** 10.1155/2019/3548917

**Published:** 2019-06-11

**Authors:** Fernanda Vieira Paladino, Juliana de Moraes Rodrigues, Aline da Silva, Anna Carla Goldberg

**Affiliations:** ^1^Hospital Israelita Albert Einstein, São Paulo, Brazil; ^2^Departamento de Alergia e Imunopatologia, Faculdade de Medicina, Universidade de São Paulo, Brazil; ^3^Instituto de Investigação em Imunologia-INCT, Brazil

## Abstract

The benefits attributed to mesenchymal stem/stromal cells (MSC) in cell therapy applications are mainly attributed to the secretion of factors, which exhibit immunomodulatory and anti-inflammatory effects and stimulate angiogenesis. Despite the desirable features such as high proliferation levels, multipotency, and immune response regulation, there are important variables that must be considered. Although presenting similar morphological aspects, MSC collected from different tissues can form heterogeneous cellular populations and, therefore, manifest functional differences. Thus, the source of MSC should be a factor to be considered in the development of novel therapies. The following text presents an updated review of recent research outcomes related to Wharton's jelly mesenchymal stem/stromal cells (WJ-MSC), harvested from umbilical cords and considered novel and potential candidates for the development of cell-based approaches. This text highlights information on how WJ-MSC affect immune responses in comparison with other sources of MSC.

## 1. Introduction

Mesenchymal stem/stromal cells (MSC) are increasingly viewed as sources of cell therapy applications due to their known immunomodulatory and anti-inflammatory effects and capacity to stimulate angiogenesis. Despite the desirable features such as high proliferation levels, multipotency, and immune response regulation, there are important variables that must be considered. Although presenting similar morphological aspects, MSC collected from different tissues can form heterogeneous cellular populations and also manifest tissue-specific functional differences. Thus, the source of MSC should be a factor to be considered in the development of novel therapies. The following text presents an updated review of recent research outcomes related to Wharton's jelly mesenchymal stem/stromal cells (WJ-MSC), harvested from umbilical cords and considered novel and potential candidates for the development of cell-based therapies. This text highlights information on how WJ-MSC affect immune responses in comparison with other sources of MSC. Some of the challenges to be addressed in order to overcome hurdles associated with the therapeutic application of these cells are also included.

## 2. The Umbilical Cord Is the Source of Wharton's Jelly

**Figure 1:**
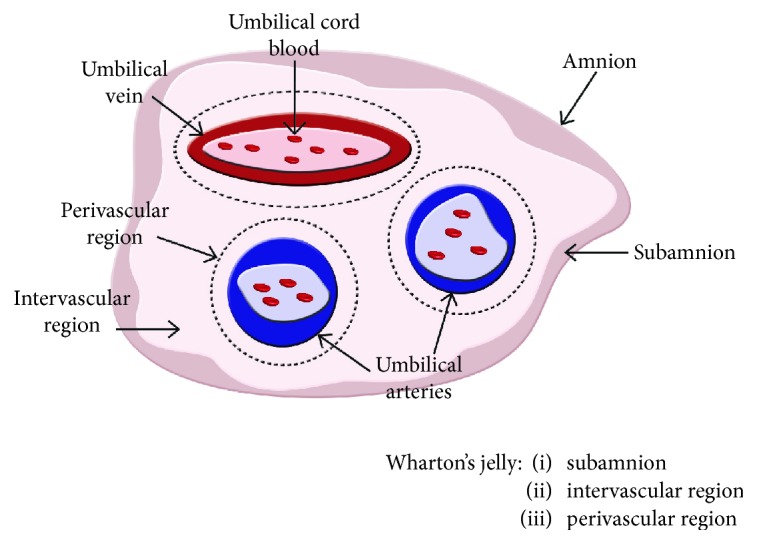
Human umbilical cord structure. Schematic image showing umbilical cord anatomical compartments, including Wharton's jelly.

Wharton's jelly (WJ) can be generally described as the mucoid connective tissue that encloses the three umbilical vessels, one vein and 2 arteries, being surrounded by a single layer of amniotic epithelial cells, which constitute the human umbilical cord [1]. Recently, the ongoing interest in umbilical cords as a useful source of MSC encouraged further investigation on these tissue structures. WJ is currently divided into three main zones based on their histological appearance: (a) the subamnion with a sparse population of fibroblast-like cells; (b) the intervascular region, a matrix of connective tissue predominantly made from collagen I, which concentrates the greatest proportion of WJ-MSC; and (c) the perivascular layer that surrounds the umbilical vessels (Figure 1) [2, 3]. WJ-MSC derived from different parts of the same umbilical cord are equally valuable sources for use in cell therapy [4]. Of note, WJ-MSC are different from the hematopoietic stem cells found in the umbilical cord blood [5]. Moreover, as other authors already described [3], WJ is seeded by distinct sources of mesenchymal/stromal cells during the embryological development. These cell subsets express not only relevant markers that characterize both WJ-MSC and perivascular cells but possibly also the main source of progenitor cells that populate the WJ [6].

## 3. Benefits of Using WJ-MSC

MSC are considered a potential tool for cell therapy. The “gold standard” bone marrow-derived MSC (BM-MSC) are the most used in clinical trials but have shown mixed results [7–12]. Furthermore, their use is not always recommended due to the techniques needed to obtain the cell. BM-MSC are isolated from bone marrow aspirate; this is an invasive procedure and painful for the patient and is accompanied by a risk of infection, possibility of donor morbidity, differences in donor age, and still change or loss of *in vitro* proliferative and differentiation cellular capacity [13, 14].

Alternative sources where isolation is easier, like adipose tissue (AT) and WJ [15], should be and are being considered. AT is an autologous source of cells though some concerns like donor age and risk of infection are the same when compared to bone marrow (BM) [16]. Other alternative sources are, for example, dental pulp [17] and menstrual blood (reviewed in [18]), a well-recognized source of MSC known since 2004. The umbilical cord is usually discarded, mitigating the risks associated with the invasive procedures needed to isolate MSC from BM [16]. With few ethical concerns, WJ is considered an easily accessible source of MSC. WJ-MSC have been compared not only with BM-MSC but also with AT-MSC (adipose tissue-MSC) and MSC derived from menstrual blood [19] and in most cases, show higher proliferative capacity. In addition, WJ-MSC are very young cells derived from a protected neonatal tissue that has suffered less environmental interference, namely, the effects on the tissues resulting from disease history and life style, a fact that helps the acquisition of a more uniform cell cohort, which may favor their therapeutic application. However, the outcome of functional tests *in vitro* indicates that they too exhibit limited lifespans and variable immune suppression potentials [20–23]. WJ-MSC are less prone to develop defective functions that can accumulate throughout a cells' lifespan due to aging and the lifetime exposure to environmental factors [24]. It is important to take into account that quality control for these cells should follow specific criteria such as selecting samples from healthy donors of full-term pregnancies, women over 18 years of age, water broken for no longer than 18h, and the expectant mother must have had at least two consultations during pregnancy and should not present fever or infection at time of birth. Maternal serum screening before delivery should include hemoglobin electrophoresis and serology for prevalent viruses and parasites.

Several reports describe MSC as immune privileged or hypoimmunogenic cells, a status likely enhanced by immunologically protected neighbouring sites, the placenta and the foetus itself [25]. In fact, they express low levels of MHC class I and costimulatory CD40, CD80, and CD86. They also lack expression of MHC class II molecules [24, 26, 27], in spite of the observation of an upregulated HLA-DR expression on BM-MSC after treatment with IFN-*γ*, but not with TNF-*α*. Nevertheless, differing from BM-MSC HLA-DR expression, the same authors did not detect the effect on WJ-MSC [28]. WJ-MSC exhibit enhanced expression of immune suppression proteins, notably leukocyte antigen G6 (HLA-G6) known to have an important role in avoiding immune-based responses against the embryo, indoleamine-2,3-dioxygenase (IDO), and prostaglandin E2 (PGE2) [29].

An important point for consideration is the fact that therapeutic applications involving MSC require an initial *in vitro* expansion step prior to their use and generally hundreds of millions of cells are used per treatment. It has been shown that several passages *in vitro* leads to a decrease in BM-MSC self-renewal capacity measured by telomere length shortening and increase in senescence markers [30]. Studies usually evaluate the immunomodulatory capacity of MSC from different sources only in early passages, and few data in the current literature is available on their behavior after passaging *in vitro* until enough numbers of cells are obtained for use in cell therapy [31, 32]. One study comparing AD-MSC and BM-MSC from passage 4 to passage 10 showed that they had similar cell morphology, surface marker expression, and immunomodulatory properties, even though gene expression was different [33]. Despite a higher lifetime *in vitro*, renewal of WJ-MSC ultimately will also lead to cell arrest and replicative senescence and the result will be the loss of stem cell functionality, even though the senescent cells remain alive [34–36]. We previously observed [20] that WJ-MSC from different donors exhibited different lifespans, as measured by senescent phenotype, number of passages, and expansion potential. Moreover, each WJ-MSC sample presented a unique behavior, differing in patterns of cytokine mRNA expression and immunomodulatory properties [37]. Thus, we believe that careful evaluation of senescence markers after repeated passaging plus monitoring of the immunosuppressant potential of each harvested cell must be included in quality control before therapeutic use.

## 4. Therapeutic Uses Based on the Immunomodulatory Effects of MSC: Comparing WJ-MSC with BM-MSC

When a tissue is damaged, inflammation occurs and tissue-resident MSC and even BM-MSC are mobilized to the lesion site [29, 38]. Because of their multipotency, it was believed that recruited MSC differentiated into functional cells to replace the damaged ones. However, this occurrence has eluded researchers. Studies using autologous cells mainly from bone marrow and adipose tissue and/or allogeneic cells from umbilical cord blood have shown that after infusion transdifferentiation of MSC into functional cells in tissues rarely occurs if at all [39]. In turn, it has become increasingly clear that in response to an inflammatory milieu, MSC prepare the microenvironment for tissue repair by producing immunoregulatory molecules that modulate the progression of inflammation, releasing growth factors to produce extracellular matrix [40], stimulating the *in situ* progenitor cells to differentiate and replace lost cells [41], and promoting angiogenesis [42]. The apparent incongruity between the benefit achieved and the lack of differentiation of the recruited MSC into specialized tissue cells has led to the unraveling of the surprising immunosuppressive capacity of MSC from many different sources [43–48].

By now, it is well known that the most promising benefits of therapy with MSC occur in patients presenting inflammatory or autoimmune diseases [49, 50]. Thus, the MSC immunomodulatory effects may play an important role in the improvement of autoimmune diseases like systemic lupus erythematosus [51, 52], type 1 diabetes mellitus [53], and multiple sclerosis [54, 55]. Ringden and Le Blanc showed that treatment using an allogenic source of MSC from umbilical cord blood (UCB-MSC), not WJ-MSC, was able to reverse partially or totally GVHD in 50% of patients [56]. In addition, the group headed by Krampera et al. [57] and other researchers [45, 58, 59] sought to unveil the immunomodulatory mechanisms of BM-MSC, confirming their effect on proliferation and antigen-specific responses by T lymphocytes.

WJ-MSC also appear to show a robust immunomodulatory potential [22]. A comparative study using MSC derived from whole human umbilical cord (MC-MSC) WJ-MSC and BM-MSC showed that MC-MSC proliferated faster and survive longer in culture than WJ-MSC; however, they have similar immunomodulatory potential [60]. Another study comparing BM-MSC and WJ-MSC demonstrated that inflammation affects the immune properties of MSC sources in different ways. Priming BM-MSC enhanced the suppression of phytohemagglutinin (PHA) mitogen-stimulated T cells only, whereas IFN-*γ*-primed WJ-MSC were better suppressors of MLR (mixed lymphocyte reaction) [28]. BM-MSC, WJ-MSC, and AT-MSC were all capable of suppressing T cell proliferation [61, 62]. However, high levels of IL-17A were detected in WJ-MSC cocultures, which is one of the key mediators in the treatment of graft-versus-host disease [61]. In a murine experimental autoimmune encephalomyelitis (EAE) model, WJ-MSC treated with IFN-*γ* increased regulatory T (Treg) cell proliferation and decreased the secretion of inflammatory cytokines in EAE mice, reducing the symptoms of the disease [63].

Of note, human fetal bone marrow (FBM-MSC) and WJ-MSC have biological advantages as compared to adult cells [62]. WJ-MSC have a gene expression pattern similar to AT-MSC but not FBM-MSC. Beyond that, genes associated with cell adhesion, proliferation, and immunomodulatory function are increased in WJ-MSC as revealed by gene ontology. WJ-MSC intrinsically overexpress genes involved in neurotrophic support when compared to BM-MSC, which makes WJ-MSC an interesting candidate for cell therapy in neurodegenerative disorders [64].

## 5. MSC Exert Comprehensive Effects on Cell-Mediated Immune Responses

MSC can interact with and regulate the activation and function of immune cells, such as T and B lymphocytes [65, 66], dendritic cells (DC) [67], and monocytes/macrophages [68]. The effects of MSC on the immune system are generally anti-inflammatory and are achieved by different, but complementary mechanisms.

Nicola et al. showed that human BM-MSC are capable of suppressing T cell proliferation in a mixed lymphocyte reaction (MLR) or when T cells are activated by phytohemagglutinin (PHA) [45]. WJ-MSC suppress mitogen-induced T cell responses to a greater extent than either BM-MSC or AT-MSC [28]. Recently, our group showed that different samples of human WJ-MSC were capable of inhibiting mitogen-activated CD3+ T cell proliferation, although to different extents, though the immunomodulatory profile of each WJ-MSC was essentially maintained even after 10 passages [37]. Another mechanism involved in immune suppression is T cell anergy. BM-MSC can induce T cell anergy by suppressing cyclin D2 expression and inhibiting CD4+ and CD8+ T cell proliferation by producing nitric oxide [69, 70]. BM-MSC are also capable of regulating the immune response by the induction of Treg, and it has been reported that they can induce T cell apoptosis via the Fas/FasL pathway. The apoptotic cells will stimulate macrophages to secrete high levels of TGF-*β*, which in turn will generate Treg cells [71]. Our preliminary results (unpublished data) showed that WJ-MSC were also able to induce Treg cells when cocultured with PBMC and treated with IFN-*γ*. BM-MSC also affect B cell functions, inhibiting the proliferation of activated B cells, their antibody production, and their chemotactic behavior [72]. BM-MSC have been shown to interfere in differentiation, maturation, and function of DC [67]. For example, in coculture, DC lose their ability to induce T cell activation [73–75]. Likewise, the differentiation of monocytes into mature DCs was inhibited and costimulatory ligand expression was blocked when cultured with WJ-MSC [76]. Taken together, the available literature indicates that WJ-MSC possess immunological features comparable to the better studied BM-MSC and even to MSC from other sources, but further detailing is needed to find the best therapeutic indications for this allogeneic source of cells as a substitute for the autologous BM-MSC and AT-MSC. The fact that WJ-MSC constitute an allogeneic therapy may in fact favor these cells in certain pathologies where the immunosuppressive response is urgent and should encompass cell and humoral responses.

An additional twist in this rationale is the observation that MSC, both *in vitro* and *in vivo*, seem capable of adopting a pro- or anti-inflammatory phenotype. Similar to the phenotype-switching phenomenon in macrophages massively explored throughout the literature and reviewed elsewhere [77, 78], MSC are also sensitive to shifts in the local immune milieu. Disruption towards an excessive concentration of proinflammatory cytokines such as IFN-*γ* and TNF-*α* activates signalling pathways by way of sensors present on human BM-MSC, causing a shift to the MSC2 phenotype and playing an important role in the downregulation of immune cells and their corresponding proinflammatory mediators [79]. In contrast, to switch to a MSC1-type profile, an anti-inflammatory microenvironment is required and MSC1 will not only express lower levels of immunosuppressive genes including IDO, NO, and PGE2 but will also be a major source of proinflammatory molecules, which will recruit and activate immune cells by secreting IL-6 and producing IL-1*α* and IL-1*β* [78]. BM-MSC, under conditions of hypoxia and stimulated with proinflammatory cytokines such as IFN-*γ*, TNF-*α*, and IL-1*β*, increase the expression of Toll-like receptors TLR2, TLR3, and TLR4, rendering these cells more sensitive to the inflammatory medium [80]. Waterman et al. showed that BM-MSC acquired two distinct phenotypes after stimulation with TLR3 and TLR4 ligands and accordingly, resulted in different immunomodulatory effects. Indeed, LPS-stimulated BM-MSC (TLR4 ligand) exhibited a proinflammatory profile (MSC1) in contrast with the polyI:C stimulated BM-MSC (TLR3 ligand) that showed an anti-inflammatory profile (MSC2) [79]. The same group also showed that BM-MSC induced into expressing the MSC1 profile attenuate cancer cell growth while when the same cells exhibit a MSC2 phenotype, they act similarly to conventional MSC in promoting tumor growth and metastases [81].

The bottom-line result of MSC switching to a type 1 profile is ultimately an overall immune modulation opposing the local environment [78]. In an inflammatory milieu, the induction of a type 2 MSC will lead to the regulation of excessive immune responses at the focal point of injury, the desirable scenario to heal damaged tissue, sponsored and facilitated by MSC plasticity.

## References

[1] Davies J. E., Walker J. T., Keating A. (2017). Concise review: Wharton’s jelly: the Rich, but enigmatic, source of mesenchymal stromal cells. *Stem Cells Translational Medicine*.

[2] Takechi K., Kuwabara Y., Mizuno M. (1993). Ultrastructural and immunohistochemical studies of Wharton’s jelly umbilical cord cells. *Placenta*.

[3] Nanaev A. K., Kohnen G., Milovanov A. P., Domogatsky S. P., Kaufmann P. (1997). Stromal differentiation and architecture of the human umbilical cord. *Placenta*.

[4] Bharti D., Shivakumar S. B., Park J. K. (2018). Comparative analysis of human Wharton’s jelly mesenchymal stem cells derived from different parts of the same umbilical cord. *Cell and Tissue Research*.

[5] Karahuseyinoglu S., Cinar O., Kilic E. (2007). Biology of stem cells in human umbilical cord stroma: in situ and in vitro surveys. *Stem Cells*.

[6] Schugar R. C., Chirieleison S. M., Wescoe K. E. (2009). High harvest yield, high expansion, and phenotype stability of CD146 mesenchymal stromal cells from whole primitive human umbilical cord tissue. *Journal of Biomedicine and Biotechnology*.

[7] Owen M. (1988). Marrow stromal stem cells. *Journal of Cell Science*.

[8] Bianco P., Riminucci M., Gronthos S., Robey P. G. (2001). Bone marrow stromal stem cells: nature, biology, and potential applications. *Stem Cells*.

[9] Arnous S., Mozid A., Mathur A. (2011). The bone marrow derived adult stem cells for dilated cardiomyopathy (REGENERATE-DCM) trial: study design. *Regenerative Medicine*.

[10] Bhansali A., Asokumar P., Walia R. (2014). Efficacy and safety of autologous bone marrow-derived stem cell transplantation in patients with type 2 diabetes mellitus: a randomized placebo-controlled study. *Cell Transplantation*.

[11] Pal R., Venkataramana N. K., Bansal A. (2009). Ex vivo-expanded autologous bone marrow-derived mesenchymal stromal cells in human spinal cord injury/paraplegia: a pilot clinical study. *Cytotherapy*.

[12] Reinders M. E. J., de Fijter J. W., Roelofs H. (2013). Autologous bone marrow-derived mesenchymal stromal cells for the treatment of allograft rejection after renal transplantation: results of a phase I study. *Stem Cells Translational Medicine*.

[13] Stenderup K., Justesen J., Clausen C., Kassem M. (2003). Aging is associated with decreased maximal life span and accelerated senescence of bone marrow stromal cells. *Bone*.

[14] Hass R., Kasper C., Böhm S., Jacobs R. (2011). Different populations and sources of human mesenchymal stem cells (MSC): a comparison of adult and neonatal tissue-derived MSC. *Cell Communication and Signaling*.

[15] Sriramulu S., Banerjee A., Di Liddo R. (2018). Concise review on clinical applications of conditioned medium derived from human umbilical cord-mesenchymal stem cells (UC-MSCs). *International Journal of Hematology-Oncology and Stem Cell Research*.

[16] Bieback K., Kern S., Kocaömer A., Ferlik K., Bugert P. (2008). Comparing mesenchymal stromal cells from different human tissues: bone marrow, adipose tissue and umbilical cord blood. *Bio-Medical Materials and Engineering*.

[17] Yazid F. B., Gnanasegaran N., Kunasekaran W., Govindasamy V., Musa S. (2014). Comparison of immunodulatory properties of dental pulp stem cells derived from healthy and inflamed teeth. *Clinical Oral Investigations*.

[18] Gargett C. E., Schwab K. E., Deane J. A. (2016). Endometrial stem/progenitor cells: the first 10 years. *Human Reproduction Update*.

[19] Ren H., Sang Y., Zhang F., Liu Z., Qi N., Chen Y. (2016). Comparative analysis of human mesenchymal stem cells from umbilical cord, dental pulp, and menstrual blood as sources for cell therapy. *Stem Cells International*.

[20] Paladino F. V., Peixoto-Cruz J. S., Santacruz-Perez C., Goldberg A. C. (2016). Comparison between isolation protocols highlights intrinsic variability of human umbilical cord mesenchymal cells. *Cell and Tissue Banking*.

[21] Amari A., Ebtekar M., Moazzeni S. M. (2015). Investigation of immunomodulatory properties of human Wharton’s jelly-derived mesenchymal stem cells after lentiviral transduction. *Cellular Immunology*.

[22] Valencic E., Piscianz E., Andolina M., Ventura A., Tommasini A. (2010). The immunosuppressive effect of Wharton’s jelly stromal cells depends on the timing of their licensing and on lymphocyte activation. *Cytotherapy*.

[23] Choo K. B., Tai L., Hymavathee K. S. (2014). Oxidative stress-induced premature senescence in Wharton’s jelly-derived mesenchymal stem cells. *International Journal of Medical Sciences*.

[24] Kalaszczynska I., Ferdyn K. (2015). Wharton’s jelly derived mesenchymal stem cells: future of regenerative medicine? Recent findings and clinical significance. *BioMed Research International*.

[25] Le Blanc K., Tammik C., Rosendahl K., Zetterberg E., Ringdén O. (2003). HLA expression and immunologic properties of differentiated and undifferentiated mesenchymal stem cells. *Experimental Hematology*.

[26] Wang M., Yang Y., Yang D. (2009). The immunomodulatory activity of human umbilical cord blood-derived mesenchymal stem cells in vitro. *Immunology*.

[27] Zhou C., Yang B., Tian Y. (2011). Immunomodulatory effect of human umbilical cord Wharton’s jelly-derived mesenchymal stem cells on lymphocytes. *Cellular Immunology*.

[28] Prasanna S. J., Gopalakrishnan D., Shankar S. R., Vasandan A. B. (2010). Pro-inflammatory cytokines, IFN*γ* and TNF*α*, influence immune properties of human bone marrow and Wharton jelly mesenchymal stem cells differentially. *PLoS One*.

[29] Weiss M. L., Anderson C., Medicetty S. (2008). Immune properties of human umbilical cord Wharton’s jelly-derived cells. *Stem Cells*.

[30] Baxter M. A., Wynn R. F., Jowitt S. N., Wraith J. E., Fairbairn L. J., Bellantuono I. (2004). Study of telomere length reveals rapid aging of human marrow stromal cells following in vitro expansion. *Stem Cells*.

[31] Izadpanah R., Kaushal D., Kriedt C. (2008). Long-term in vitro expansion alters the biology of adult mesenchymal stem cells. *Cancer Research*.

[32] Stolzing A., Jones E., McGonagle D., Scutt A. (2008). Age-related changes in human bone marrow-derived mesenchymal stem cells: consequences for cell therapies. *Mechanisms of Ageing and Development*.

[33] Mun C. H., Kang M. I., Shin Y. D., Kim Y., Park Y. B. (2018). The expression of immunomodulation-related cytokines and genes of adipose- and bone marrow-derived human mesenchymal stromal cells from early to late passages. *Tissue Engineering and Regenerative Medicine*.

[34] Yang Y. H. K., Ogando C. R., Wang See C., Chang T. Y., Barabino G. A. (2018). Changes in phenotype and differentiation potential of human mesenchymal stem cells aging in vitro. *Stem Cell Research & Therapy*.

[35] Khong S. M. L., Lee M., Kosaric N. (2019). Single-cell transcriptomics of human mesenchymal stem cells reveal age-related cellular subpopulation depletion and impaired regenerative function. *Stem Cells*.

[36] Itahana K., Dimri G., Campisi J. (2001). Regulation of cellular senescence by p53. *European Journal of Biochemistry*.

[37] Paladino F. V., Sardinha L. R., Piccinato C. A., Goldberg A. C. (2017). Intrinsic variability present in Wharton’s jelly mesenchymal stem cells and T cell responses may impact cell therapy. *Stem Cells International*.

[38] Hoogduijn M. J., Popp F., Verbeek R. (2010). The immunomodulatory properties of mesenchymal stem cells and their use for immunotherapy. *International Immunopharmacology*.

[39] Wang Y., Chen X., Cao W., Shi Y. (2014). Plasticity of mesenchymal stem cells in immunomodulation: pathological and therapeutic implications. *Nature Immunology*.

[40] Yang Y., Lin H., Shen H., Wang B., Lei G., Tuan R. S. (2018). Mesenchymal stem cell-derived extracellular matrix enhances chondrogenic phenotype of and cartilage formation by encapsulated chondrocytes in vitro and in vivo. *Acta Biomaterialia*.

[41] Prockop D. J., Kota D. J., Bazhanov N., Reger R. L. (2010). Evolving paradigms for repair of tissues by adult stem/progenitor cells (MSCs). *Journal of Cellular and Molecular Medicine*.

[42] Xu L., Zhou J., Liu J. (2017). Different Angiogenic potentials of mesenchymal stem cells derived from umbilical artery, umbilical vein, and Wharton’s jelly. *Stem Cells International*.

[43] Meesuk L., Tantrawatpan C., Kheolamai P., Manochantr S. (2016). The immunosuppressive capacity of human mesenchymal stromal cells derived from amnion and bone marrow. *Biochemistry and Biophysics Reports*.

[44] Manochantr S., U-pratya Y., Kheolamai P. (2013). Immunosuppressive properties of mesenchymal stromal cells derived from amnion, placenta, Wharton’s jelly and umbilical cord. *Internal Medicine Journal*.

[45] Di Nicola M., Carlo-Stella C., Magni M. (2002). Human bone marrow stromal cells suppress T-lymphocyte proliferation induced by cellular or nonspecific mitogenic stimuli. *Blood*.

[46] Hong J. W., Lim J. H., Chung C. J. (2017). Immune tolerance of human dental pulp-derived mesenchymal stem cells mediated by CD4^+^CD25^+^FoxP3^+^ regulatory T-cells and induced by TGF-*β*1 and IL-10. *Yonsei Medical Journal*.

[47] Mohammadi Ayenehdeh J., Niknam B., Rasouli S. (2017). Immunomodulatory and protective effects of adipose tissue-derived mesenchymal stem cells in an allograft islet composite transplantation for experimental autoimmune type 1 diabetes. *Immunology Letters*.

[48] Luz-Crawford P., Torres M. J., Noël D. (2016). The immunosuppressive signature of menstrual blood mesenchymal stem cells entails opposite effects on experimental arthritis and graft versus host diseases. *Stem Cells*.

[49] Le Blanc K., Frassoni F., Ball L. (2008). Mesenchymal stem cells for treatment of steroid-resistant, severe, acute graft-versus-host disease: a phase II study. *The Lancet*.

[50] Németh K., Leelahavanichkul A., Yuen P. S. T. (2009). Bone marrow stromal cells attenuate sepsis via prostaglandin E_2_–dependent reprogramming of host macrophages to increase their interleukin-10 production. *Nature Medicine*.

[51] Sun L., Akiyama K., Zhang H. (2009). Mesenchymal stem cell transplantation reverses multiorgan dysfunction in systemic lupus erythematosus mice and humans. *Stem Cells*.

[52] Sun L., Wang D., Liang J. (2010). Umbilical cord mesenchymal stem cell transplantation in severe and refractory systemic lupus erythematosus. *Arthritis & Rheumatism*.

[53] Mesples A., Majeed N., Zhang Y., Hu X. (2013). Early immunotherapy using autologous adult stem cells reversed the effect of anti-pancreatic islets in recently diagnosed type 1 diabetes mellitus: preliminary results. *Medical Science Monitor*.

[54] Karussis D., Karageorgiou C., Vaknin-Dembinsky A. (2010). Safety and immunological effects of mesenchymal stem cell transplantation in patients with multiple sclerosis and amyotrophic lateral sclerosis. *Archives of Neurology*.

[55] Freedman M. S., Bar-Or A., Atkins H. L. (2010). The therapeutic potential of mesenchymal stem cell transplantation as a treatment for multiple sclerosis: consensus report of the International MSCT Study Group. *Multiple Sclerosis*.

[56] Ringden O., Le Blanc K. (2011). Mesenchymal stem cells for treatment of acute and chronic graft-versus-host disease, tissue toxicity and hemorrhages. *Best Practice & Research Clinical Haematology*.

[57] Krampera M., Glennie S., Dyson J. (2002). Bone marrow mesenchymal stem cells inhibit the response of naive and memory antigen-specific T cells to their cognate peptide. *Blood*.

[58] Le Blanc K. (2003). Immunomodulatory effects of fetal and adult mesenchymal stem cells. *Cytotherapy*.

[59] Aggarwal S., Pittenger M. F. (2005). Human mesenchymal stem cells modulate allogeneic immune cell responses. *Blood*.

[60] Mennan C., Brown S., McCarthy H. (2016). Mesenchymal stromal cells derived from whole human umbilical cord exhibit similar properties to those derived from Wharton’s jelly and bone marrow. *FEBS Open Bio*.

[61] Karaöz E., Çetinalp Demircan P., Erman G., Güngörürler E., Eker Sarıboyacı A. (2017). Comparative analyses of immunosuppressive characteristics of bone-marrow, Wharton’s jelly, and adipose tissue-derived human mesenchymal stem cells. *Turkish Journal of Haematology*.

[62] Wang Q., Yang Q., Wang Z. (2016). Comparative analysis of human mesenchymal stem cells from fetal-bone marrow, adipose tissue, and Warton’s jelly as sources of cell immunomodulatory therapy. *Human Vaccines & Immunotherapeutics*.

[63] Torkaman M., Ghollasi M., Mohammadnia-Afrouzi M., Salimi A., Amari A. (2017). The effect of transplanted human Wharton’s jelly mesenchymal stem cells treated with IFN-*γ* on experimental autoimmune encephalomyelitis mice. *Cellular Immunology*.

[64] Donders R., Bogie J. F. J., Ravanidis S. (2018). Human Wharton’s jelly-derived stem cells display a distinct immunomodulatory and proregenerative transcriptional signature compared to bone marrow-derived stem cells. *Stem Cells and Development*.

[65] Le Blanc K., Rasmusson I., Gotherstrom C. (2004). Mesenchymal stem cells inhibit the expression of CD25 (interleukin-2 receptor) and CD38 on phytohaemagglutinin-activated lymphocytes. *Scandinavian Journal of Immunology*.

[66] Uccelli A., Moretta L., Pistoia V. (2006). Immunoregulatory function of mesenchymal stem cells. *European Journal of Immunology*.

[67] Jiang X. X., Zhang Y., Liu B. (2005). Human mesenchymal stem cells inhibit differentiation and function of monocyte-derived dendritic cells. *Blood*.

[68] Cho D. I., Kim M. R., Jeong H. Y. (2014). Mesenchymal stem cells reciprocally regulate the M1/M2 balance in mouse bone marrow-derived macrophages. *Experimental & Molecular Medicine*.

[69] Glennie S., Soeiro I., Dyson P. J., Lam E. W., Dazzi F. (2005). Bone marrow mesenchymal stem cells induce division arrest anergy of activated T cells. *Blood*.

[70] Ren G., Zhang L., Zhao X. (2008). Mesenchymal stem cell-mediated immunosuppression occurs via concerted action of chemokines and nitric oxide. *Cell Stem Cell*.

[71] Akiyama K., Chen C., Wang D. D. (2012). Mesenchymal-stem-cell-induced immunoregulation involves FAS-ligand-/FAS-mediated T cell apoptosis. *Cell Stem Cell*.

[72] Corcione A., Benvenuto F., Ferretti E. (2006). Human mesenchymal stem cells modulate B-cell functions. *Blood*.

[73] Zhang W., Ge W., Li C. (2004). Effects of mesenchymal stem cells on differentiation, maturation, and function of human monocyte-derived dendritic cells. *Stem Cells and Development*.

[74] Nauta A. J., Kruisselbrink A. B., Lurvink E., Willemze R., Fibbe W. E. (2006). Mesenchymal stem cells inhibit generation and function of both CD34^+^-derived and monocyte-derived dendritic cells. *The Journal of Immunology*.

[75] Spaggiari G. M., Abdelrazik H., Becchetti F., Moretta L. (2009). MSCs inhibit monocyte-derived DC maturation and function by selectively interfering with the generation of immature DCs: central role of MSC-derived prostaglandin E_2_. *Blood*.

[76] Tipnis S., Viswanathan C., Majumdar A. S. (2010). Immunosuppressive properties of human umbilical cord-derived mesenchymal stem cells: role of B7-H1 and IDO. *Immunology and Cell Biology*.

[77] Bunnell B. A., Betancourt A. M., Sullivan D. E. (2010). New concepts on the immune modulation mediated by mesenchymal stem cells. *Stem Cell Research & Therapy*.

[78] Bernardo M. E., Fibbe W. E. (2013). Mesenchymal stromal cells: sensors and switchers of inflammation. *Cell Stem Cell*.

[79] Waterman R. S., Tomchuck S. L., Henkle S. L., Betancourt A. M. (2010). A new mesenchymal stem cell (MSC) paradigm: polarization into a pro-inflammatory MSC1 or an immunosuppressive MSC2 phenotype. *PLoS One*.

[80] Raicevic G., Rouas R., Najar M. (2010). Inflammation modifies the pattern and the function of Toll-like receptors expressed by human mesenchymal stromal cells. *Human Immunology*.

[81] Waterman R. S., Henkle S. L., Betancourt A. M. (2012). Mesenchymal stem cell 1 (*MSC1*)-based therapy attenuates tumor growth whereas *MSC2*-treatment promotes tumor growth and metastasis. *PLoS One*.

